# Annual acknowledgement of manuscript reviewers

**DOI:** 10.1186/1756-0500-7-35

**Published:** 2014-01-16

**Authors:** Christopher Foote

**Affiliations:** 1 BioMed Central, 236 Gray’s Inn Road, London WC1X 8HB, UK

## Abstract

**Contributing reviewers:**

The editors of BMC Research Notes would like to thank all of our reviewers who have contributed to the journal in volume 6 (2013).

## 

Mette Aadahl

Denmark

Fatemeh Abbasalizadeh

Iran

Alaa Abd-Elsayed

Egypt

Amina Abubakar

Netherlands

Maria Czarina Acelajado

Philippines

Olivia Achonduh

Cameroon

Ishag Adam

Sudan

David Adamson

USA

Cheryl Addy

Qatar

Anthony Ayodeji Adegoke

South Africa

Isaac Adewole

Nigeria

Wichai Aekplakorn

Thailand

Amitesh Aggarwal

India

Agoston Agoston

USA

Tahera Ahmed

Bangladesh

Avan Aihie Sayer

UK

Beyza Akdag

Turkey

Bassel Al-Alao

UK

Sinaa Alaqeel

Saudi Arabia

Teresa Alarcon

Spain

Steven Albert

USA

Marco Albonico

Italy

Abdel Aziem Ali

Sudan

Ahmed Ali

Egypt

Amir Ali

Saudi Arabia

Ahmet Alim

Turkey

Thomas Alter

Germany

Susana Alvarez

Argentina

Najith Amarasena

Australia

Srikanth Ambati

USA

Richard Amenyah

Burkina Faso

Neda Aminzadeh

Iran

Satoshi Anai

Japan

Gashaw Andargie

Ethiopia

Christina Ander

Germany

Bjørg Marit Andersen

Norway

Johan Hviid Andersen

Denmark

Mikael Rørdam Andersen

Denmark

Irina Anderson

UK

M Brownell Anderson

USA

Melissa Anderson

USA

Stuart Anderson

UK

Jan Andersson

Sweden

Karl Andersson

Sweden

Heitor Andrade

Brazil

Meliala Andreasta

Indonesia

Muna Anjum

UK

Hifzur Ansari

Saudi Arabia

Pierre-Olivier Antoine

France

Tor Anvik

Norway

Hiroaki Aoyama

Japan

Desmond Archer

UK

Fabienne Archer

France

Nazli Arda

Turkey

Julija Armalyte

Lithuania

Josiane Arnaud

France

Ankur Arora

India

Narendra Kumar Arora

India

Gustavo Arrizabalaga

USA

Joanne Arsenault

USA

Atilla Arslanoglu

Turkey

Mohd Ashraf

India

Yoshimasa Aso

Japan

Stelios Assimakopoulos

Greece

Matias Attene Ramos

USA

Alexandra Aubry

France

Linda Avesani

Italy

Asfar Azmi

USA

Tetsuro Baba

Japan

Lorena Baccaglini

USA

Carolyn Baglole

Canada

Quan Bai

China

Yu Zuo Bai

China

Rafik Balti

Tunisia

Jangu Banatvala

UK

Muhammad Yasin Bandukda

Pakistan

Heejung Bang

USA

Sripal Bangalore

USA

Ancha Baranova

USA

Joaquin Barnoya

Guatemala

Helena Barreto Henriksson

Sweden

J.J. Bax

Singapore

Joel Bazira

Uganda

John Beck

USA

Alexander Becker

Germany

Giorgio Bedogni

Italy

Jeffrey Bell

USA

Inna Belyantseva

USA

Yeshambel Belyhun

Ethiopia

Roberta Benetti

Italy

Jagadish Bentur

India

Gabriela Berg

Argentina

James Berger

USA

Junaid Bhatti

France

Fantahun Biadglegne

Germany

Gretchen Birbeck

USA

Luigi Bisceglia

Italy

Karl-Dimiter Bissig

USA

Marcio Sommer Bittencourt

USA

Glenn Björklund

Sweden

Giuseppe Blaiotta

Italy

Hans Rudolf Bloch

Switzerland

Jon Block

USA

Guido Bloemberg

Switzerland

Herve Blottiere

France

Mukund Bodhankar

India

Gregory Bogdanis

Greece

Ajay Bommareddy

USA

Piet Borst

Netherlands

Jean Francois Bosset

France

Xavier Bossuyt

Belgium

Ana Botelho

Portugal

Nicolle Boumans

Netherlands

Dianne Bowskill

UK

Kayvan Bozorgmehr

Germany

Salvatore Bozzaro

Italy

Sidney Braman

USA

Janet Bray

Australia

Eva Broström

Sweden

Nicholas Brown

USA

Richard Brown

UK

Glenn Browning

Australia

Robb Brumfield

USA

Teerapong Buaboocha

Thailand

Hikmet Budak

Turkey

Rebecca Bunnell

Kenya

Peter Burbelo

USA

Irene Burckhardt

Germany

Shawn Burgess

USA

Andreas Burkovski

Germany

Sakib Burza

India

Azeez Butali

USA

Alida Caforio

Italy

Demetria Cain

USA

Ronan Canavan

Ireland

Rossella Canese

Italy

Stuart Cantsilieris

Australia

Vincenzo Cardinale

Italy

Alexander Cardoso

Brazil

Mark A. Carlson

USA

Vera Carneiro

Brazil

Silvana Carnevale

Argentina

Emerson Carraro

Brazil

Thierry Carrel

Switzerland

Andres Carrillo

Greece

Luis Carvalho

USA

Rita Casadio

Italy

Leandro Castellano

UK

John Castle

Germany

Antonio Castro

Brazil

Philippe Cattin

Switzerland

Aaron Caughey

USA

Silvia Cavalcanti

Brazil

Corrado Cecchetti

Italy

Mauro Cervigni

Italy

Montakarn Chaikumarn

Thailand

Anna Chaimani

Greece

Ching-Wei Chang

USA

Jui-Hsing Chang

Taiwan

Anand Chaudhary

India

Iskander Chaudhry

UK

Vijay Chava

India

Nikolaos Chavouzis

Greece

Chien-Yu Chen

Taiwan

Guo-Lin Chen

USA

Li Chen

USA

Liang Chen

China

Hao-Min Cheng

Taiwan

Ming Chi

USA

Ming-Chou Chiang

Taiwan

Matteo Chiappedi

Italy

Carla Chibwesha

Zambia

Violet Chihota

South Africa

Rayan Chikhi

France

Chandeshwari Chilampalli

USA

Andrew Childs

UK

Jerome Chin

USA

Chin Chiun-Li

Taiwan

Young June Choe

South Korea

Wen-Chi Chou

USA

Gerardo Chowell

USA

Bo Young Chun

South Korea

Daniela M Cirillo

Italy

Damian Clarke

South Africa

Greg Cook

New Zealand

Robin Corelli

USA

Ricardo Correa

USA

David Cosgrove

UK

Eduardo Costa

Brazil

Mark Cotton

South Africa

Jean Tenena Coulibaly

Cote d'Ivoire

Mick Couper

USA

Patrice Courvalin

France

Chris Cousens

UK

Benoit Couvigny

France

Nicholas Cowans

UK

Lenore Cowen

USA

Benjamin Cowling

Hong Kong

Vasile Cozma

Romania

Holger Cramer

Germany

Mary Cramp

UK

John Craven

Australia

Lory Saveria Crocè

Italy

Geert Crombez

Belgium

Jane Cross

UK

Fuqiang Cui

China

Juan Cui

USA

Antonio Curra

Italy

Anna Cywinska

Poland

Alda Maria Da-Cruz

Brazil

Hannah Dada-Adegbola

Nigeria

Giuseppe D'Agostino

Italy

Maria Cristina D'Agostino

Italy

Neetu Dahiya

USA

Vankudoth Dal Singh

India

Jeanette Daly

USA

Jesse Damsker

USA

Hussein Daood

Hungary

Monidipa Dasgupta

Canada

Agnelli Davide

Italy

Luiz Carlos De Abreu

Brazil

Pier Giuseppe De Benedetti

Italy

Etienne Declercq

Belgium

Abraham Degarege

Ethiopia

Rosa Del Angel

Mexico

Thomas Deleuran

Denmark

Romano Demicheli

Italy

Lutfiye Demir

Turkey

Omer Demir

Turkey

Yucel Demiral

Turkey

Sharon Demorrow

USA

Ayele Denboba

Italy

Ning Deng

China

Rajeev Desai

USA

Olufemi Desalu

Nigeria

Kassu Desta

Ethiopia

Efstathios Detorakis

Greece

Moyez Dharsee

Canada

Govinda Dhungana

Nepal

Vidal Díaz de Rada

Spain

Marco Dibonaventura

USA

Julia Diegelmann

Germany

Ioannis Dimitroulis

Greece

Petros Dinas

Greece

Wei-Qun Ding

USA

Konstantina Dipla

Greece

Mamuka Djibuti

Georgia

Bien-Cuong Do

Vietnam

Jane Dobson

UK

Michael J. Doenhoff

UK

Simon Donell

UK

Horng-Yunn Dou

Taiwan

Scott Dowell

USA

Dennis Dowhan

Australia

Finn Drabløs

Norway

Bedis Dridi

Canada

Mignon du Plessis

South Africa

Le Duc Hau

South Korea

Jorge Duconge

Puerto Rico

Tim Dudderidge

UK

Andrew Duncan

USA

Julie Dunning Hotopp

USA

Rajasekhara reddy Duvvuru Muni

USA

Hang-Korng Ea

France

Zoe Edelstein

USA

Orestis Efthimiou

Greece

Tim Eglinton

New Zealand

Emmanuel Ejim

Nigeria

Ibrahim Elghissassi

Morocco

Magdy El-Salhy

Norway

Solomon Emishaw

Ethiopia

Tobias Erb

Switzerland

Koray Ergunay

Turkey

Joachim Ernst

Germany

Aydan Eroglu

Turkey

Annette Kjaer Ersboell

Denmark

Shankar Esaki Muthu

Malaysia

Josiane Etang

Cameroon

Neal Evenhuis

USA

Sophie Evison

UK

Cynthia Fabian-Madrazo

Philippines

Joseph Fadare

Nigeria

Omar Faiz

UK

Vicenç Falcó

Spain

Paul Falkowski

USA

Éanna Falvey

Ireland

Kuangnan Fang

China

Cathy Sj Fann

Taiwan

Yang Fann

USA

Joao Faria

Portugal

Jocelyn Faubert

Canada

Emmanuel Favaloro

Australia

Kurt Fellenberg

Germany

Richelle Jeannette Fyline Felt

Netherlands

Yifan Feng

China

Matthew Ferrari

USA

Luciana Ferraro

Italy

Gudrun Feuchtner

Austria

Jean Feuillard

France

Marc G.J. Feuilloley

France

Carolina Fierro

Belgium

Jose Figueroa

UK

Lionel G Filion

Canada

Nicholas Finer

UK

Jeffrey Finnie

South Africa

Francesca Finotello

Italy

Aryeh Fischer

USA

Peter Fischer

USA

Phil Fischer

USA

Horace Fletcher

Jamaica

Virginia Fonner

USA

Andrew Hugh Ford

Australia

Claire Foster

UK

Marie Foulongne-Oriol

France

Johnson Francis

India

Octavio Franco

Brazil

Andrew Franklyn-Miller

Australia

Thomas Friedl

Germany

Rogerio Friedman

Brazil

Holger Fröhlich

Germany

Toshinobu Fujiwara

Japan

Martha Funnell

USA

Reema Gabrani

India

Daniel Gale

UK

Madhavi Ganapathiraju

USA

Maria Ganczak

Poland

Rohan Ganguli

Canada

Feng Gao

China

Enrico Garattini

Italy

Frederic Garban

France

Paola Gargiulo

Italy

Akanksha Garud

India

Gasim Gasim

Sudan

Laurent Gatto

UK

Abhishek Gaur

India

Maria Gazouli

Greece

Endrias Gebremedhin

Ethiopia

Gavin Lloyd George

South Africa

Ekoyol Ewane Germaine

Cameroon

Constance Gewa

USA

Maibritt Giacobini

Sweden

Christoforos Giannaki

Cyprus

Georgios Giannakoulas

Greece

Fiseha Girma

Ethiopia

Saulius Girnius

USA

David Gisselquist

USA

Daniele Giuffrida

Italy

Cinzia Giuli

Italy

Sofia Gkountela

USA

Shannon Glaser

USA

N. Louise Glass

USA

Shimon Glick

Israel

Pieter Goeminne

Belgium

Richard Goering

USA

Björn Gohlke

Germany

George Golding

Canada

Susan Goldstein

USA

Majid Golkar

Iran

Rodolfo Gomez

UK

Tanja Gonska

Canada

Debbie Goode

UK

Daniel Gordin

Finland

Andrew Goudie

UK

Shakti Gounder

Fiji

Evanthia Gouveri

Greece

Navin Goyal

USA

Tomaz Gracner

Slovenia

Mike Gray

UK

Esther Green

Canada

Ted Greiner

South Korea

Aurelien Grellet

France

Ulla Griffiths

UK

Luciane Grillo

Brazil

Grazyna Gromadzka

Poland

Ivan Guerra

Spain

Joao Guerreiro

Brazil

Paul Guest

UK

Dilek Guldal

Turkey

Rong Guo

China

Amit Gupta

India

Sudesh Gyawali

Nepal

Jessica Haberer

USA

Daniela Christina Hadorn

Switzerland

Lukman Hakim

Belgium

Douglass Hale

USA

Alix Hall

Australia

Peter Hall

Brazil

Sang-Uk Han

South Korea

Yuyan Han

USA

Sukwan Handali

USA

Johanna Hänggi

Switzerland

Thomas Hänscheid

Portugal

Sebastien Hantz

France

Ross Hardison

USA

Kim Hare

Australia

Sudharshan Hariharan

USA

Hacer Harlak

Turkey

Guy Harling

USA

Lynda Harris

UK

Ingunn Harstad

Norway

Peter Harvey

Kenya

Rumina Hasan

Pakistan

Ziad Hassan

Hungary

Angelos Hatzakis

Greece

Stephen Hawes

USA

Kazutoshi Hayase

Japan

Ping-An He

China

Rong-Qiao He

China

Martin Helmkampf

USA

Marleen Hendriks

Netherlands

Christiane Herden

Germany

Matthias Heringlake

Germany

Victoria Hernando

Spain

Thomas Heyse

Germany

Hiroshi Hiasa

USA

Alicia Hidalgo

UK

Riet Hilhorst

Netherlands

D. Ashley Hill

USA

Darryl Hill

UK

Michael Hiller

Germany

Valeria Hirschler

Argentina

Jane Hislop

UK

Robert Hoffman

USA

Holly Hogrefe

USA

Carol Holm-Hansen

Norway

Rodney Honeycutt

USA

Lan T. Ho-Pham

Vietnam

Olaf Horstick

Switzerland

Guy Howard

UK

Chih-Hung Hsu

Taiwan

Dongsheng Hu

China

Guang Hu

China

Kuo-How Huang

Taiwan

Rolf Hubmayr

USA

Arthur Huen

USA

David Humphreys

Australia

Robert Hurst

USA

Tahziba Hussain

India

Ming-Jing Hwang

Taiwan

Emmanuel Idigbe

Nigeria

Alexander Idnurm

USA

Akira Iguchi

Japan

Caio Imaizumi

Brazil

Shinsaku Imashuku

Japan

Nicholas Ingolia

USA

Giles Innocent

UK

Pablo Irimia

Spain

Seth Irish

UK

Karin E Isaksson Ro

Norway

Nobuhisa Ishikawa

Japan

Syazwan Aizat Ismail

Malaysia

Yasuhiro Ito

Japan

Hilde Hestad Iversen

Norway

Ali Izadpanah

Canada

Arnaud Jaccard

France

Felice Jacka

Australia

Shalini Jadia

India

Ilesh Jani

Mozambique

Bertrand Janne d'Othee

USA

Tavan Janvilisri

Thailand

Jin-Tsong Jeng

Taiwan

Edmund Jenkins

USA

Subrina Jesmin

Bangladesh

Dar-Der Ji

Taiwan

Steinar Johansen

Norway

Dinesh John

USA

Jeff Johnson

USA

Mallory Johnson

USA

Natalie Johnson

Australia

Kate Jolly

UK

Graham Jones

Australia

Kurt Jordaens

Belgium

Farahnaz Joukar

Iran

Abdo Jurjus

Lebanon

Ali Kabir

Iran

S. M. Lutful Kabir

Bangladesh

Christos Kalaitzis

Greece

Spyridon Kampantais

Greece

Tanuj Kanchan

India

Eva Johanna Kantelhardt

Germany

Neena Kanwar

USA

Christian Kappel

Germany

Ayse Demet Karaman

Turkey

Mehran Karimi

Iran

Samuel Kariuki

Kenya

Monika Kastner

Canada

Masayuki Kato

Japan

Melissa Kaufman

USA

Pankaj Kaushal

India

Yoshiaki Kawamura

Japan

Masakado Kawata

Japan

Patrick Kayembe

Democratic Republic of Congo

Edmund Kayombo

Tanzania

Erin Kelleher

USA

George Kenna

USA

Andrew Kerkhoff

USA

Ken Kesler

USA

Muhammad Asim Khan Rehmani

Pakistan

Mahboobeh Khorsandi

Iran

Paramjit Khurana

India

Meehyein Kim

South Korea

Ruth Kimokoti

USA

Lesley King

Jamaica

Craig Kinnear

South Africa

Jefferson Kinney

USA

Marit Kirkevold

Norway

Philippe Kiss

Belgium

Kenichiro Kitamura

Japan

Steen Knudsen

Denmark

Martin Koebel

Canada

Ruchika Kohli

Kenya

Manabu Koike

Japan

George Konstantinou

USA

Mirjam Kooistra-Smid

Netherlands

Trond M Kortner

Norway

Selda Koydemir

Turkey

Tomoaki Kozaki

Japan

Avdyl Krasniqi

Serbia

Guha Krishnaswamy

USA

Bjarni Kristjansson

Iceland

Birgit Kroener-Herwig

Germany

David Krpata

USA

Senanayake Kularatne

Sri Lanka

Hemant Kulkarni

USA

Ambuj Kumar

USA

Sanjay Kumar

India

Hsien-Wen Kuo

Taiwan

Phillip Kuo

USA

Motoyasu Kusano

Japan

Stepan Kutilek

Czech Republic

Alexander Kuznetsov

Germany

Susanne La Fleur

Netherlands

Eliana Lacerda

UK

Dirk Lachenmeier

Germany

Salil Lachke

USA

Benoit Lacombe

France

Philippe Lagacé-Wiens

Canada

Eliane Lages-Silva

Brazil

Pauline Siew Mei Lai

Malaysia

Marta Lana

Brazil

Paul Langan

USA

Malinee Laopaiboon

Thailand

Lidia Larizza

Italy

Mette Larsen

Denmark

Mauricio Lascano

USA

Larry Latson

USA

Dominique Lavenier

France

Vladimir Lazarevic

Switzerland

Jeffrey Lazarus

Denmark

Thomas Le Corroller

France

Rosario Le Moli

Italy

Olivier Le Saux

USA

Andrew Leask

Canada

Bong Hwa Lee

South Korea

Seonghee Lee

USA

Dink Legemate

Netherlands

Sandra Leggat

Australia

Tiago Leiria

Brazil

Jasna Lenicek Krleza

Croatia

Wilma Leslie

UK

Miriam Levy

Australia

Els Leye

Belgium

Dinorah Leyva

USA

Guoan Li

USA

Xia Li

China

Xin Li

USA

Liunn-Wang Liao

Taiwan

Eleni Liapi

USA

Alan Lill

Australia

Sandra Maria Lima Ribeiro

Brazil

Daniel Limonta

Cuba

Hung-Yin Lin

Taiwan

Wen-Yuan Lin

Taiwan

Barbro Linderholm

Sweden

Lars Lindner

Germany

Jacqueline Lindo

UK

Hui Ling

USA

Alexander Link

Germany

Patricia Lisboa

Brazil

Mikhail Lisovsky

USA

Baogang Liu

China

Jie Liu

China

Munghua Liu

China

Tsunglin Liu

Taiwan

Yongchao Liu

Germany

Zheng Liu

USA

Callum Livingstone

UK

Attilio Ignazio Lo Monte

Italy

Thurmon Lockhart

USA

Angela Lombardi

Italy

Quanxin Long

China

Arne Lowden

Sweden

Chang-Tien Lu

USA

Chuanjian Lu

China

Xinghua Lu

USA

Yue Lu

USA

Viraphong Lulitanond

Thailand

Jennifer Lund

Denmark

Karen Luxford

Australia

Jeremy Lynch

USA

Lut Lynen

Belgium

Una Macintyre

South Africa

Anca Macovei

Romania

Irene Madrigal

Spain

Eduardo Madrigal-Bujaidar

Mexico

Nicola Maffiuletti

Switzerland

Luca Maggio

Italy

Edson Maistro

Brazil

Mwai Makoka

Malawi

Christina Malavaki

Greece

Gathsaurie Malavige

Sri Lanka

Ketil Malde

Norway

Sarmila Mallik

India

James Malone Lee

UK

Ouattara Mamadou

Cote d'Ivoire

Justin Mandala

USA

Martin Mangino

USA

Antoni Manolis

Greece

Loaie Maraqa

UK

Christian Marcelli

France

Giorgos Margaritopoulos

Greece

Maria Maridaki

Greece

Guillermo Andrés Maroniche

Argentina

Mario Marques

Portugal

Pier Luigi Martelli

Italy

Andrew Martin

Australia

Karen Martin

USA

Carmen Martin-Cordero

Spain

Jose Maria Martin-Duran

Norway

Martínez Martínez

Spain

Miguel Martinez-Gonzalez

Spain

Maria Conceicao Martins

Brazil

Elizabeth Marum

USA

Marinko Marusic

Croatia

Marco Marzioni

Italy

Linnet Masese

USA

Saidur Mashreky

Bangladesh

Simona Masiero

Italy

John Mason

USA

Philip Mason

USA

Biniam Mathewos

Ethiopia

Sandra Mathioni

Brazil

Gladys Motlagabo Matseke

South Africa

Akira Matsuno

Japan

Luciano Mavilia

Italy

Anthony Maxwell

UK

Claus Mayer

UK

Oscar Mayer

USA

Shali Mazaki-Tovi

USA

Kelly McDaniel

USA

Sheena McGowan

Australia

Pam McGrath

Australia

Sarah McIntyre

Australia

Geoff McLachlan

Australia

Matthew McMillin

USA

James McNair

UK

Elizabeth McPherson

USA

Takafira Mduluza

Zimbabwe

Jesús L. Megías

Spain

Andreas Mentis

Greece

Adalberto Merighi

Italy

George Metsios

UK

Francois Meurens

Canada

Valeria Mezzolla

Italy

William Miller

USA

Snezana Milutinovic

USA

Joanna Milward

UK

Clare Minahan

Australia

Joel Mindel

USA

Masahiro Mishina

Japan

Kate Mitchell

UK

Mariya Miteva

USA

Lampros Mitrakas

Greece

Thimios Mitsiadis

Switzerland

Omprakash Mittapalli

USA

Norimasa Miura

Japan

Spiros Miyakis

Australia

Toru Mizuguchi

Japan

Zalilah Mohd Shariff

Malaysia

Alex Molassiotis

Hong Kong

Barbara Molesini

Italy

Attila Molvarec

Hungary

Christodoulos Monastiriotis

Greece

Maurizio Mongia

Italy

Rosa Monno

Italy

Ali Montazeri

Iran

Ginny Moore

UK

Sarah Moore

UK

Krishna Moorthy

UK

Gabriel Moreno-Hagelsieb

Canada

Susan Morpeth

Kenya

Giulia Morsica

Italy

Sara Mostafalou

Iran

Miguel Moyses Neto

Brazil

Wendy Mphatswe

South Africa

Anita Mukherjee

India

Kimberly Murray

USA

Laurent Musango

Congo

Kim Musser

USA

Nora Mutalima

UK

Adamson Muula

Malawi

Joseph Mwangangi

Kenya

Shun-Ichi Nagata

Japan

Yusuf Nagree

Australia

Eliana Aguiar Petri Nahas

Brazil

Hidetomo Nakamoto

Japan

Yoji Nakamura

Japan

George Nakos

Greece

Darren Natale

USA

Heda Melinda Nataprawira

Indonesia

Benjamin Natelson

USA

Gerardo Nava

USA

Rafael Navajas-Pérez

Spain

Fina Navi

Iran

Giesje Nefs

Netherlands

Ben Nephew

USA

Ines Neves

Portugal

Maria New

USA

Cecilia Ng

Australia

Brian Nguyen

USA

Van Huy Nguyen

Vietnam

Julie Nightingale

UK

Nobuo Niimura

Japan

Dimitrios Nikolopoulos

Greece

Lena Maria Nilsson

Sweden

Veeranoot Nissapatorn

Malaysia

Peter Nissen

Denmark

Hiroaki Nitta

USA

Angelika A. Noegel

Germany

Tsering Norboo

India

Caroline Norez

France

Angela Notarnicola

Italy

Paolo Nucci

Italy

Paulo Nuin

Canada

Marco Nunes

Brazil

Maria Isabel Nuñez

Spain

Uchenna Nwagha

Nigeria

Thomas O'Brien

USA

J.G. Ocejo-Vinyals

Spain

Ifedayo Odeniyi

Nigeria

Aoife O'Donovan

USA

Tufan Oge

Turkey

Paul O'Grady

Ireland

Tagbo Oguonu

Nigeria

Yuki Ohsaki

Japan

Mervi Oikonen

Finland

German Ojeda-Correal

USA

Henrietta Uchenna Okafor

Nigeria

Chijioke Okoli

Nigeria

Charles Okwundu

South Africa

Marlon Olimulder

Netherlands

Adelaide Olson

USA

Albert Oriol

Spain

Tareq Osaili

Jordan

Mohamed Osman

Egypt

Mahmoudreza Ovissipour

USA

Richard Owczarzy

USA

Volkan Özenci

Sweden

Serdar Oztora

Turkey

Luis Pacheco

Brazil

Gennaro Pagano

Italy

Kathleen Page

USA

Tiziana Pandolfini

Italy

Georgios Panos

Switzerland

Antonios Papadopoulos

Greece

Evangelia Papakonstanti

Greece

Evridiki Papastavrou

Cyprus

Konstantinos Papatheodorou

Greece

Athanasia Papazafiropoulou

Greece

Valentina Parisi

Italy

Yung Park

South Korea

Laurie Parker

USA

Marco Passamonti

Italy

Antonio Luigi Pastore

Italy

Ravi Patel

USA

Victor Patterson

UK

James Patton

USA

Giulio Pavesi

Italy

John Pearson

New Zealand

Alma Becic Pedersen

Denmark

Susanne Bendesgaard Pedersen

Denmark

Maria Lucia Penna

Brazil

Jennifer Perera

Sri Lanka

Gastón Perman

Argentina

Wei Perng

USA

Fabien Pernot

France

Patrick Petignat

Switzerland

Kelley Pettee Gabriel

USA

Susanne Pettersson

Sweden

John Pettifor

South Africa

Adrian Philbey

UK

Tom Pickles

Canada

Armin Piehler

Norway

Prisco Piscitelli

Italy

M. Dolors Piulachs

Spain

Benjamin Piura

Israel

Audrey Player

USA

Palmiro Poltronieri

Italy

Loganathan Ponnusamy

USA

Todd Porter

USA

Emmanuel Pothos

USA

Shinu Pottathil

India

Ian Poxton

UK

Scott Pratt

USA

David Preston

USA

Are Hugo Pripp

Norway

Paolo Provero

Italy

Evangelos Psomas

Greece

Basant Puri

UK

Manju Purohit

India

Ibrahim Qaddoumi

USA

Rui-Qun Qi

China

Bradley Quade

USA

Stuart Quan

USA

Philip Quanjer

Netherlands

Vasilios Raftopoulos

Cyprus

Shadab Rahman

USA

Om Rajora

Canada

Dhamodharan Ramasamy

France

Jayashree Ramasethu

USA

Jose Manuel Ramos

Spain

Surinder Rana

India

Latha Rangan

India

Mahesh Rao

India

Debolina Ray

USA

Daniel Rayson

Canada

Serena Redaelli

Italy

Raja Reddy

UK

Ramalinga Reddy

USA

Josep Redon

Spain

Marvin Reid

Jamaica

Ulf Reineke

Germany

Bernhard Renard

Germany

Dirk Repsilber

Germany

Oleg Reva

South Africa

Amelia Ribeiro De Jesus

Brazil

Henry Rice

USA

Kelly Rice

USA

Laetitia Rispel

South Africa

Diogo Rivelli

Brazil

David Roberts

UK

Oscar Rocha

USA

Cecilia Rodriguez

Mexico

Kristien Roelens

Belgium

Claire Rogel-Gaillard

France

Michael Roizen

USA

Hamilton Roschel

Brazil

Anna Rose

UK

Pernilla Roswall

Sweden

Eric Rouchka

USA

Salvatore Rubino

Italy

Bhimappa Rudagi

India

Enrique Ruiz De Gopegui

Spain

Andrew Rycroft

UK

Markku Ryynanen

Finland

Sreedharan Nair Sabarinath

USA

Ulrich Sack

Germany

Ashis Saha

India

Sudipto Saha

India

Salah Said

Netherlands

Shilpakala Sainath Rao

USA

Lesley Ann Saketkoo

USA

Mateus Sakundarno

Indonesia

Carlos Salas

Brazil

Raquel Sa-Leao

Portugal

Siba Samal

USA

Victor Sanchez-Margalet

Spain

Anna Sannino

Italy

Sreevidya Santha

USA

Valter Santilli

Italy

Alfredo Santovito

Italy

Bishwa Sapkota

Japan

Stefania Sarno

Italy

Takashi Sasaki

Japan

Tatiana Sato

Brazil

Nongyao Sawangjaroen

Thailand

John Sayer

UK

Thomas Scalea

USA

Frieder Schaumburg

Germany

Enrico Schleiff

Germany

Heiko Schmidt

Austria

Manfred Schmitt

Germany

Roland Schmitt

Germany

Laura Schnackenberg

USA

Nicole Schupf

USA

Marta Scorsetti

Italy

Andrzej Sechman

Poland

Teresa Seefeldt

USA

Laure Segurel

USA

Andrada Seicean

Romania

Hanna Seidling

Germany

Yoshika Sekine

Japan

Masashi Sekino

Japan

Salaam Semaan

USA

Efraim Serafetinides

Greece

Zineb Serhier

Morocco

Graham Serjeant

Jamaica

Ahmad Settin

Saudi Arabia

Gavin Sewell

UK

Chiarella Sforza

Italy

Juliet Shaffer

USA

Muamar Shaheen

Palestinian Territory

Vikal Shakya

Nepal

Nathan Shankar

USA

Ning-Yi Shao

USA

Adam Shapiro

USA

Deepak Sharan

India

Tsu-Wang Shen

Taiwan

Arthur Chun-Chieh Shih

Taiwan

Takahito Shikano

Finland

Akira Shimatsu

Japan

Techalew Shimelis

Ethiopia

Norikazu Shimizu

Japan

Tatsuo Shimosawa

Japan

Seiji Shiota

Japan

Akiko Shiotani

Japan

Drlivy Shivraj

India

Steve Shochat

USA

Noam Shomron

Israel

Noam Shomron

USA

Natalia Shubladze

Georgia

Ketia Shumaker

USA

Abhinav Sidana

USA

Monica Sierra

France

Jose Sifuentes-Osornio

Mexico

Rolf Sijmons

Netherlands

Mauro Silvestrini

Italy

Mark Simmerman

Thailand

Pouth Simon Franky

Cameroon

Petros Skapinakis

Greece

Vesna Skodric Trifunovic

Serbia

Elizabeth Skovran

USA

Alena Skrahina

Belarus

Hans-Christian Slotved

Denmark

Guy Smagghe

Belgium

Sophia Smith

USA

Lars Snipen

Norway

John Snowdon

Australia

Rodrigo Soares

Brazil

Jean-Karl Soler

Malta

Bettina Sommer

Germany

Joon Jin Song

USA

Smita Sood

India

Joseph Sorg

USA

Peter Sörös

Canada

Manuel Soto

Spain

Maria Elena Soto

Mexico

Francisco Soto Mas

USA

Ivan Soto-Calderon

Colombia

Paul Span

Netherlands

Peter Spencer

USA

Silvana Spinelli

Argentina

Anand Srivastava

USA

Richard Stabler

UK

Lisa Staten

USA

Antonios Stavropoulos-Kalinoglou

Greece

Aggelos Stefos

Greece

Martin Steinberg

USA

Timothy Steiner

Norway

Melinda Steis

USA

Christine Stirling

Australia

Barbara Stoecker

USA

Margaret Stone

UK

William Stone

USA

Daniel Stuckey

UK

Robert Stupar

USA

Jackie Sturt

UK

Wei-Juin Su

Taiwan

Yoshinori Sugiura

Japan

Myung-Whan Suh

South Korea

Jae Hoon Sul

USA

Carolyn Summerbell

UK

Anne Sumner

USA

Fengjie Sun

USA

Zhonghua Sun

Australia

Prabhu Sundararaman

India

Fred Sundström

Sweden

Alastair Sutcliffe

UK

Iain Sutcliffe

UK

Esther Suter

Canada

Madeline Sutton

USA

Yasuhiko Suzuki

Japan

Elisabeth Svensson

Sweden

Olga Szafran

Canada

Grace Szeto

Hong Kong

Yt Szeto

Hong Kong

Alfred Szmidt

Japan

Benjamin Szymanski

USA

Takafumi Tabuchi

Japan

Rosna Taha

Malaysia

Aki Takahashi

Japan

Howard Takiff

Venezuela

Flora Tassone

USA

Belaynew Wasie Taye

Ethiopia

James Taylor

USA

John Taylor

USA

Takele Teklu

Ethiopia

Petra Temming

Germany

Nandor Gabor Than

USA

Seenivasagan Thangaraj

India

Rajat Thawani

India

Theodoros Theodoridis

Greece

Eric Thomas

USA

Charlotte Thomas-Hawkins

USA

William Thompson

USA

Bruce Thorley

Australia

Kamala Thriemer

Belgium

Thibault Thubert

France

Claudio Tiribelli

Italy

Marcello Tiseo

Italy

Kelvin To

Hong Kong

Thomas Tompkins

Canada

Louis Tong

Singapore

Laura Toppino

Italy

Stefano Torriani

Switzerland

Balagangadhar Totapally

USA

Michael Toth

USA

John Tower

USA

Jeffrey Townsend

USA

Loraine Townsend

South Africa

Thach Tran

Vietnam

Lyndal Trevena

Australia

Vladimir Trifonov

Russia

Isheng Jason Tsai

UK

Iraklis Tsangaris

Greece

Alexandra Tsigginou

Greece

Abigail Tucker

UK

Ayse Sibel Turkum

Turkey

Paul Turner

Cambodia

Anna Tylki-Szymanska

Poland

Argyris Tzouvelekis

Greece

Mariko Uehara

Japan

Glen Ulett

Australia

Yoshihisa Urita

Japan

Yavuz Uyar

Turkey

Edi Vaisbuch

USA

Mariano J. Valderrama

Spain

Angelica Valente

Italy

Olga van den Akker

UK

Willem van der Does

Netherlands

Dimitri van der Linden

Netherlands

Sacha van Hijum

Netherlands

Brian van Wyk

South Africa

David Vance

USA

Luiz Carlos Marques Vanderlei

Brazil

M Pilar Vaquero

Spain

Carlo Vascotto

Italy

Gilberto Vaughan

USA

Pradeep Venkatesh

India

Lucio Vera-Cabrera

Mexico

Francisco J Vera-Garcia

Spain

Marina Vercelli

Italy

Petra Verdonk

Netherlands

Jan Vermorken

Belgium

David Verner-Jeffreys

UK

John Vetto

USA

Tapani Viitala

Finland

Kunjupillai Vijayan

India

Torstein Vik

Norway

Kris Vissenberg

Belgium

G.L. Viswanatha

India

Maria Paulina Viteri Espinel

USA

Panagiotis Vlachostergios

Greece

David Vogel

USA

Victoriya Volkova

USA

Ludovica Volpi

Italy

Arndt von Haeseler

Austria

Neil Vora

USA

Gert Vriend

Netherlands

Mogens Vyberg

Denmark

Indra Vythilingam

Malaysia

Juddy Wachira

USA

Elizabeth Wager

UK

Mihir Wagh

USA

Glenn Walker

Australia

Hongmei Wang

China

Nian Wang

USA

Quan Wang

USA

Weidong Wang

USA

Zhanfeng Wang

China

Selina Ward

Australia

Jon Warner

USA

Robert Weaver

UK

Shannon Weigum

USA

Elisabet Welin Henriksson

Sweden

Mary Westbrook

Australia

Gert P. Westert

Netherlands

Christopher Whelan

USA

James Whelan

Australia

Stefan White

Australia

Christophe Wiart

Malaysia

Cornelia Wiegand

Germany

Nick Wierckx

Germany

C. Mel Wilcox

USA

Arnold Wilkins

UK

Patricia Wilkins

USA

Field Willingham

USA

Thomas Willnow

Germany

Charles Wiysonge

South Africa

Arnon Wiznitzer

Israel

Eric Wong

USA

Li Ping Wong

Malaysia

Craige Wrenn

USA

Hermann Wrigge

Germany

Charlotte Wright

UK

Edward Wright

USA

Hao Wu

USA

Jaw-Ching Wu

Taiwan

Jun Wu

China

Li Xia

USA

Xiao-Yi Xiao

USA

Lei Xu

China

Tang Xudong

China

Tomohito Yagi

Japan

Genoveva Yague

Spain

Binnaz Yalcin

Switzerland

Ozlem Yalcin Tok

Turkey

Tamaki Yamada

Japan

Kunihiro Yamagata

Japan

Yasumasa Yamamoto

Japan

Kenichiro Yamamura

Japan

Can Yang

USA

Jinn-Moon Yang

Taiwan

Soo-Jin Yang

USA

Tianbing Yang

USA

Xinning Yang

USA

Bo Yao

China

Ana Patricia Yatsuda

Brazil

Veli Yazisiz

Turkey

Dorothy Yeboah-Manu

Ghana

Neville Yeomans

Australia

Li Yi

China

Kader Yildiz

Turkey

Gizachew Yismaw

Ethiopia

Efdal Yoeruek

Germany

Simon Yolung

Canada

Masa-Aki Yoshida

Japan

Hiroshi Yotsuyanagi

Japan

Hiroya Yurimoto

Japan

Leo Zacharski

USA

Anita Zamboni

Italy

Sotirios G. Zarogiannis

Greece

Endalew Zemene

Ethiopia

Mason Zhang

USA

Qiwei Zhang

China

Rui Zhang

USA

Qing Zhao

China

Xin-Qing Zhao

China

Kai Zheng

USA

Xiangqian Zheng

China

Shiliang Zhou

China

Fei Zhu

China

Eleni Zografos

Greece

Flavio Zolessi

Uruguay

Wen Zou

USA

Siti Zullaikah

Indonesia

